# Supplementary material for: Novel Driver Strength Index highlights important cancer genes in TCGA PanCanAtlas patients
Source: PeerJ. 2022 Aug 11;10:e13860. doi: 10.7717/peerj.13860 (PMC9375969; doi:10.7717/peerj.13860)
Supplement: Supplemental Information 2 [file peerj-10-13860-s002.zip › COHORTS/genes plots/detailed/2021_8_16_14_9_distribution_events_detailed_19.pdf]

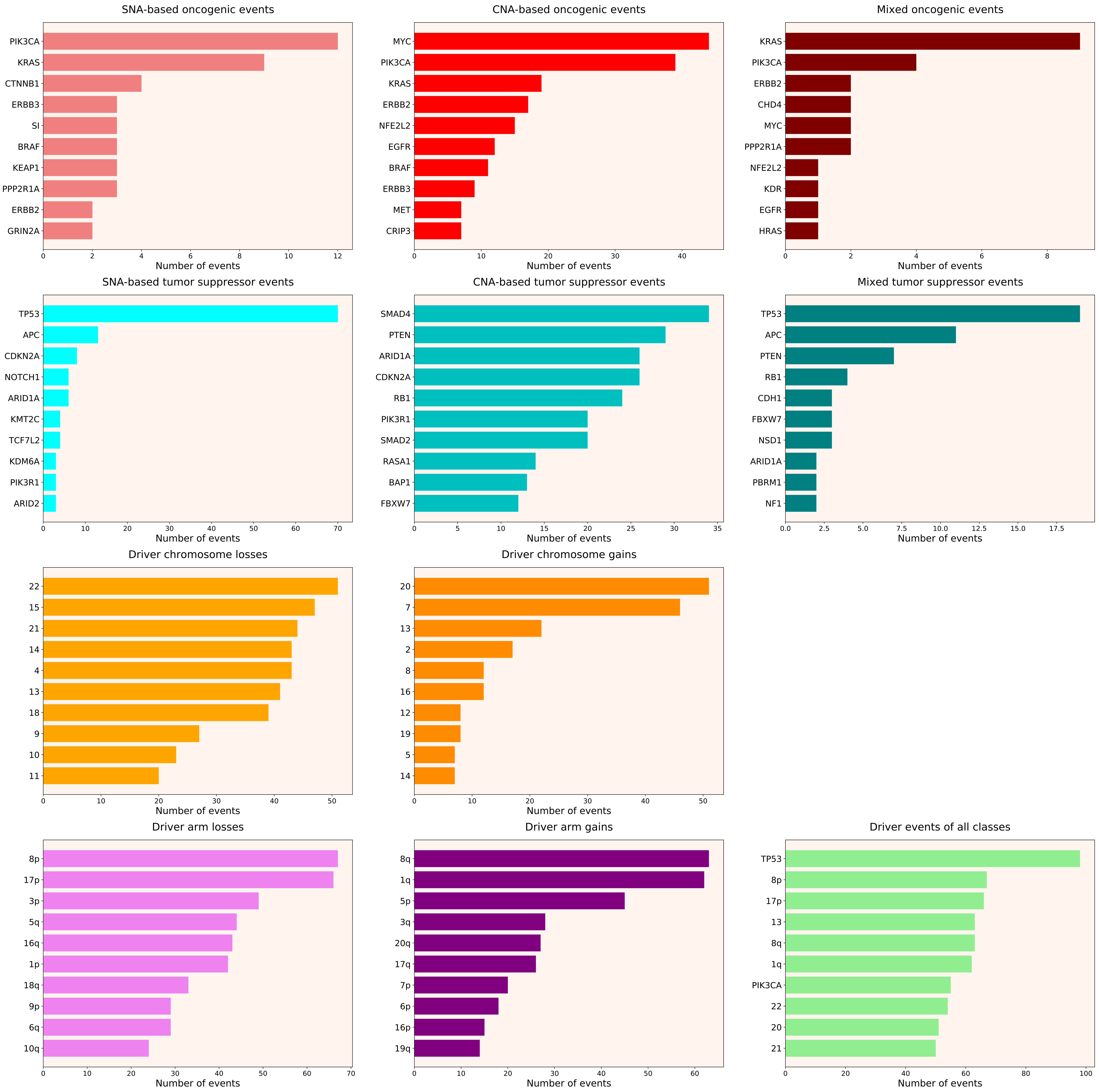
SNA-based oncogenic events
PIK3CA
KRAS
CTNNB1
ERBB3
SI
BRAF
KEAP1
PPP2R1A
ERBB2
GRIN2A
Number of events
CNA-based oncogenic events
MYC
PIK3CA
KRAS
ERBB2
NFE2L2
EGFR
BRAF
ERBB3
MET
CRIP3
Number of events
Mixed oncogenic events
KRAS
PIK3CA
ERBB2
CHD4
MYC
PPP2R1A
NFE2L2
KDR
EGFR
HRAS
Number of events
SNA-based tumor suppressor events
TP53
APC
CDKN2A
NOTCH1
ARID1A
KMT2C
TCF7L2
KDM6A
PIK3R1
ARID2
Number of events
CNA-based tumor suppressor events
SMAD4
PTEN
ARID1A
CDKN2A
RB1
PIK3R1
SMAD2
RASA1
BAP1
FBXW7
Number of events
Mixed tumor suppressor events
TP53
APC
PTEN
RB1
CDH1
FBXW7
NSD1
ARID1A
PBRM1
NF1
Number of events
Driver chromosome losses
22
15
21
14
4
13
18
9
10
11
Number of events
Driver chromosome gains
20
7
13
2
8
16
12
19
5
14
Number of events
Driver arm losses
8p
17p
3p
5q
16q
1p
18q
9p
6q
10q
Number of events
Driver arm gains
8q
1q
5p
3q
20q
17q
7p
6p
16p
19q
Number of events
Driver events of all classes
TP53
8p
17p
13
8q
1q
PIK3CA
22
20
21
Number of events
